# CCR6, the Sole Receptor for the Chemokine CCL20, Promotes Spontaneous Intestinal Tumorigenesis

**DOI:** 10.1371/journal.pone.0097566

**Published:** 2014-05-27

**Authors:** Bisweswar Nandi, Christine Pai, Qin Huang, Rao H. Prabhala, Nikhil C. Munshi, Jason S. Gold

**Affiliations:** 1 Research Service, VA Boston Healthcare System, West Roxbury, Massachusetts, United States of America; 2 Harvard Medical School, Boston, Massachusetts, United States of America; 3 Pathology, Service, VA Boston Healthcare System, West Roxbury, Massachusetts, United States of America; 4 Dana-Farber Cancer Center, Boston, Massachusetts, United States of America; 5 Medicine Service, VA Boston Healthcare System, West Roxbury, Massachusetts, United States of America; 6 Surgery Service, VA Boston Healthcare System, West Roxbury, Massachusetts, United States of America; 7 Brigham and Women's Hospital, Boston, Massachusetts, United States of America; COCHIN INSTITUTE, Institut National de la Santé et de la Recherche Médicale, France

## Abstract

Interactions between the inflammatory chemokine CCL20 and its receptor CCR6 have been associated with colorectal cancer growth and metastasis, however, a causal role for CCL20 signaling through CCR6 in promoting intestinal carcinogenesis has not been demonstrated *in vivo*. In this study, we aimed to determine the role of CCL20-CCR6 interactions in spontaneous intestinal tumorigenesis. CCR6-deficient mice were crossed with mice heterozygous for a mutation in the adenomatous polyposis coli (APC) gene (APC^MIN/+^ mice) to generate APC^MIN/+^ mice with CCR6 knocked out (CCR6KO-APC^MIN/+^ mice). CCR6KO-APC^MIN/+^ mice had diminished spontaneous intestinal tumorigenesis. CCR6KO-APC^MIN/+^ also had normal sized spleens as compared to the enlarged spleens found in APC^MIN/+^ mice. Decreased macrophage infiltration into intestinal adenomas and non-tumor epithelium was observed in CCR6KO-APC^MIN/+^ as compared to APC^MIN/+^ mice. CCL20 signaling through CCR6 caused increased production of CCL20 by colorectal cancer cell lines. Furthermore, CCL20 had a direct mitogenic effect on colorectal cancer cells. Thus, interactions between CCL20 and CCR6 promote intestinal carcinogenesis. Our results suggest that the intestinal tumorigenesis driven by CCL20-CCR6 interactions may be driven by macrophage recruitment into the intestine as well as proliferation of neoplastic epithelial cells. This interaction could be targeted for the treatment or prevention of malignancy.

## Introduction

Cytokines likely play a key role in the crosstalk between neoplastic cells and the inflammatory milieu. Chemokines are a family of small (8–10 kDa) cytokines with shared structural characteristics that are most known for directing the migration and homing of immune cells. Chemokines also play a crucial role in shaping host immunity through their involvement in processes such as the formation of lymphoid organs, wound healing, angiogenesis, and inflammation [1], [2]. Recently, it has been suggested that chemokines can play a role in cancer development and metastasis [1], [3].

Chemokines exert their effects when recognized by G-protein coupled cell surface chemokine receptors. Chemokine receptors often recognize several chemokines, and chemokines often bind to multiple receptors [1]–[3]. The C-C chemokine CCL20, also known as macrophage infiltrating factor protein-3 α (MIP-3 α) and liver activation regulated chemokine (LARC), however, is the only known ligand for the receptor CCR6, which in turn is the only known receptor for CCL20 [4]. CCR6-CCL20 interactions have been shown to be involved in several autoimmune and inflammatory processes such as rheumatoid arthritis, [5] multiple sclerosis [6], [7], psoriasis [8], and inflammatory bowel disease [9]. Of note, inflammation is particularly important in sporadic intestinal tumorigenesis [10]–[15].

The expression of CCL20 and CCR6 has been documented in several tumor types including colorectal cancer [16]–[22]. Furthermore, it has been shown that CCL20-CCR6 interactions promote increased proliferation [23], [24] and invasion [23] in colorectal cancer cell lines. The aim of this study was to assess the role of CCL20-CCR6 interactions in a mouse model of intestinal tumorigenesis.

## Materials and Methods

### Ethics statement

Written patient consent was obtained for collection of specimens. The experimental protocol was approved by the VA Boston Healthcare System Institutional Review Board (IRB). Animal experiments in this study were carried out in strict accordance with the recommendations in the Guide for the Care and Use of Laboratory Animals of the National Institutes of Health. The experimental protocol was approved by the VA Boston Healthcare System Institutional Animal Care and Use Committee (IACUC) (Protocol #291-W-111009).

### Human samples

Tissue samples were acquired immediately after resection from patients having surgery for colorectal cancer at the VA Boston Healthcare System.

### Animals

APC^MIN/+^ mice (that express one allele of a mutant adenomatous polyposis coli (APC) gene with a premature stop codon) [25], [26], CCR6 knockout (CCR6KO) mice (B6.129P2-*Ccr6^tm1Dgen^*/J), and wild-type (WT) C57/Bl6 mice were purchased from The Jackson Laboratory (Bar Harbor, ME). APC^MIN/+^ and CCR6KO mice were intercrossed to generate CCR6KO-APC^MIN/+^ and CCR6HET-APC^MIN/+^ (CCR6^+/−^-APC^MIN/+^) mice. The genotype was confirmed by performing PCR on DNA from tail snips using primers and conditions provided by the Jackson Laboratory. The mice were housed and maintained in the specific pathogen-free (SPF) animal research facility of VA Boston Healthcare System.

### Cell lines

The murine colon cancer cell line MC38 [27] was kindly provided by Michael T. Lotze of the University of Pittsburgh. The human colon cancer cell lines HT29 and Hct116 were purchased from American Type Culture Collection (ATCC; Manassas, VA). Cell lines were grown in RPMI 1640 (Life Technologies, Grand Island, NY) with 10% fetal bovine serum (FBS) (Life Technologies).

### Quantification of polyps

After euthanasia, intestines were dissected from the mice. The intestines were flushed with cold phosphate buffered saline (PBS) to remove fecal material, and then cut into three equal length segments: duodenum, jejunum and ileum. Each segment was then cut open longitudinally and examined under a dissection microscope at 10X magnification. Polyps were counted and measured at their greatest diameter. The polyp mass was defined as the sum of the greatest diameters for each polyp.

### Immunohistochemistry

Tissue rolls of each segment of mouse intestine were fixed in 10% formalin and then embedded in paraffin. Five micron tissue sections were cut and mounted on glass slides. Hematoxylin and eosin stains were performed for reference. Immunohistochemical staining was performed as described in the Dako EnVision kit (Dako North America, Inc, Carpinteria, CA) instruction manual and as detailed in the Methods S1. The primary antibodies utilized were anti-mouse B220 (clone-RA3-6B2, BD Biosciences, San Jose, CA), anti-mouse CD3 (Clone-SP7, Abcam, Cambridge, MA), anti-mouse F4/80 (clone-A3-1, AbD Serotec US, Raleigh, NC), anti-mouse FoxP3, (clone- FJK-16, eBioscience, San Diego, CA), anti-human CD163 (clone-10D6, Vector Laboratories Burlingame, CA), anti-human CCR6 (clone- 53103, R&D Systems, Minneapolis, MN), anti-human CCL20 (Polyclonal Goat IgG, R&D Systems). Cells were quantified by manual counting of 10 40X fields by a single blinded observer.

### ELISA

Snap frozen sections of mouse intestine or of human tissue were stored at −80°C until use. Homogenates were prepared either using the Tissue Lyser II (Qiagen, Valencia, CA) in a volume of 200 µl of PBS or using an Eppendorf micropestle in 1 ml Eppendorf tubes (Eppendorf, Hauppauge, NY) with 200 µl of PBS. The homogenized samples were then centrifuged at 13,000 rpm for 10 minutes to remove debris, and the supernatant was collected for analysis. Protein concentration was measured using the Quick Start Bradford protein assay (Bio-Rad Life Sciences, Hercules, CA). CCL20 levels in human or mouse tissue homogenates or in human or mouse cell culture supernatants were measured using ELISA kits for human or mouse CCL20 (R&D Systems) following the manufacturer's protocol.

### Western blot analysis

[LOOSESTCells were lysed using NP40 cell lysis buffer (Life Technologies). Cell lysates were separated by SDS-PAGE using a precast 4–15% gel (Bio-Rad, Hercules, CA) and blotted on nitrocellulose membranes (Bio-Rad, Hercules, CA) that were then washed with Tris-buffered saline (TBS) and blocked with fat-free milk in the same buffer for 1 hour at room temperature. The membranes were subject to incubation with a primary rat monoclonal antibody directed against mouse CCR6 (clone CKR-6 (13Q7), dilution 1∶200) or a mouse anti-β-actin antibody (clone ACTBD11B7, dilution 1∶1000) (both from Santa Cruz Biotechnology, Inc., Santa Cruz, CA) overnight at 4°C. The following day, the membranes were washed and incubated with goat anti-rat IgG-HRP conjugated secondary antibody (for anti-CCR6 primary antibody) and secondary goat anti-mouse IgG-HRP (for anti-β-actin primary antibody) (both at 1∶5000 dilution, Santa Cruz Biotechnology, Inc.). Finally, the membranes were developed by using the Amersham ECL Plus kit (GE Healthcare, Piscataway, NJ) as per the manufacturer's protocol.

### Immunofluorescence

2×10^5^ MC38 cells were allowed to adhere in 2 chambered Lab-Tek 4.2 cm^2^ slides (Thermo Scientific, Rochester, NY) overnight. The next day, the cells were washed with PBS and blocked with donkey serum (1∶100 dilution in PBS, Millipore, Billerica, MA) for 2 hours. Next, the cells were washed with PBS and then incubated with rat anti-mouse CCR6 monoclonal antibody (clone CKR-6 (13Q7), dilution 1∶100, Santa Cruz Biotechnology, Inc.) and polyclonal rabbit anti-cytokeratin antibody (dilution 1∶500, Dako) for 1.5 hours. The cells were washed again with PBS and then incubated with the secondary antibodies Cy5 goat anti-rat IgG and Alexa Fluor 546 goat anti-rabbit IgG antibody (both from Life Technologies) at 5 µg/ml in PBS for 1 hour. The cells were then washed and stained with SYBR Green (dilution 1∶50,000, Life Technologies) for 10 minutes. Finally, the slides were washed with PBS and air dried. The sections were sealed with a coverslip using Vectashield mounting media (Vector Laboratories) and examined using a confocal microscope.

### MTT assay

The Vybrant MTT Cell Proliferation MTT Assay Kit (Life Technologies) was used according to the manufacturer's instructions. In brief, 5×10^3^ MC38, HT29 or Hct116 cells were cultured in 100 µl of RPMI media without serum and without phenol red (Life Technologies) for 24 or 48 hours in 96 well cell culture plates. After incubation, the media was replaced with 100 µl of fresh media, and then 10 µl of 12 mM MTT solution (component A) was added to each culture well. The cells were then incubated for 4 hours at 37°C. Next, 100 µl of the SDS-HCl solution (component B) was added to each well, and the cells were incubated for another 12 hours. After incubation, the samples were mixed well, and absorbance was quantified at 570 nm using a spectrophotometer.

### RNA extraction and semi-quantitative RT-PCR

Total RNA was extracted either from tumor tissue of colorectal cancer patients or from cultured MC38, HT29 or Hct116 cells using the RNAeasy Plus Universal Kit (Qiagen, Valencia, CA). Before extracting total RNA, cell lysates were prepared from 100 mg of tumor tissue samples by mixing with 900 ml of QIAzol Lysis Reagent (Qiagen) in an Eppendorf tube followed by homogenization using the Tissue Lyser II (Qiagen). For making cell lysates from MC38, HT29 and Hct116 cells, 2×10^5^ cells were mixed with 1 ml of the QIAzol Lysis Reagent. For cDNA synthesis, 1 µg of total RNA samples were reverse-transcribed using the SuperScript III First-Strand Synthesis System (Life Technologies) where oligo-dT was used as first synthesis primer. Semi-quantitative RT-PCR for *CCR6* was performed on cDNA prepared from patient tumor tissue. The primers [28] and conditions are detailed in the Methods S1.

### Statistical analysis

Differences between means were assessed using the two-tailed Student's unpaired t-test. Differences in values for paired samples were assessed using the Wilcoxon signed-rank test. A p value <0.05 was considered significant.

## Results

### CCL20 and CCR6 are overexpressed in human colon cancer

**Figure 1:**
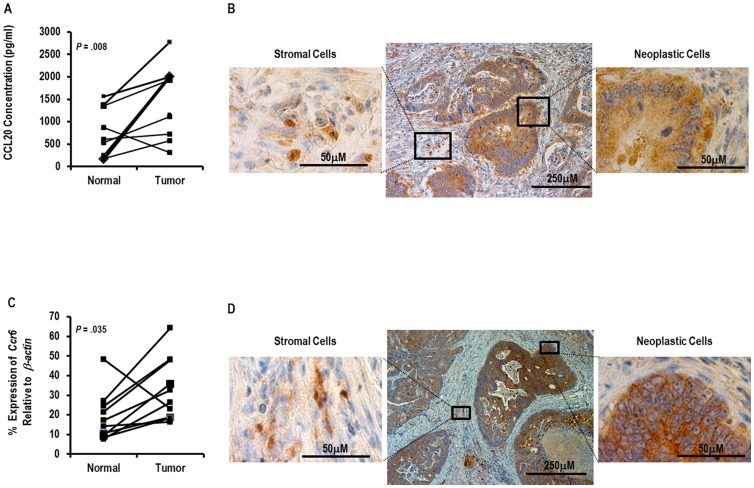
Expression of CCL20 and CCR6 in colon cancer. (A) CCL20 concentration was assayed by ELISA in 12 matched samples of colon cancer and adjacent normal colon tissue after homogenizing approximately 100 mg of tissue into 100 µl PBS. The Wilcoxon signed-rank test was used to assess significance. (B) Representative photomicrograph of a section of paraffin-embedded human colon cancer tumor tissue immunohistochemically stained for CCL20. The regions in the squares are shown magnified. (C) Relative expression of human *Ccr6* is compared in 10 matched samples of colon cancer and adjacent uninvolved colon tissue as measured by semiquantitative RT-PCR. (D) Representative photomicrograph of a section of paraffin-embedded human colon cancer tumor tissue immunohistochemically stained for CCR6. The regions in the squares are shown magnified.

To assess for an association of CCL20 and its receptor CCR6 with colorectal cancer, we evaluated the expression of CCL20 and CCR6 in human tumors. Levels of CCL20 were measured in matched samples of colorectal cancer and adjacent uninvolved colon by ELISA. While the levels of CCL20 in both non-diseased colonic tissue and colon cancer varied substantially between patients, a significant increase of CCL20 was observed in the tumors compared to the matched controls (Figure 1A). We next sought to determine the cells in the tumor microenvironment that express CCL20. Human colon cancers immunohistochemically stained for CCL20 showed high to moderate expression of CCL20 in the malignant epithelial cells in all 11 patient samples (data not shown), however, expression of CCL20 by infiltrating stromal cells was also noted (Figure 1B). Expression of CCR6, the receptor for CCL20 in human colorectal tumors and adjacent uninvolved colon was measured by semi quantitative RT-PCR (Figure 1C). Relative expression of *Ccr6* normalized to the house-keeping gene *β-actin* was compared between normal and tumor tissue. *Ccr6* was found to be expressed at a higher level in tumor tissue as compared to adjacent uninvolved normal tissue. Similar to CCL20, as determined by immunohistochemistry, CCR6 was expressed at high to moderate levels by malignant epithelial cells and to a lesser degree by infiltrating stromal cells (Figure 1D).

### CCR6KO-APC^MIN/+^ mice develop fewer intestinal adenomas than APC^MIN/+^ mice with intact CCR6

**Figure 2:**
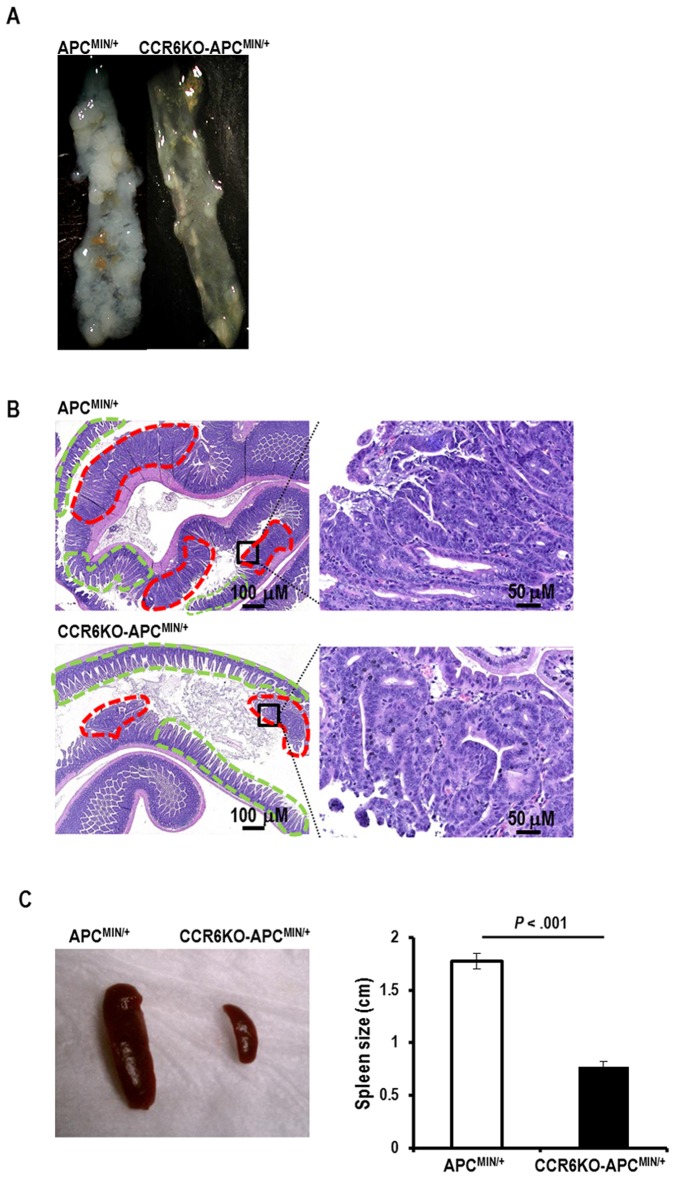
CCR6KO-APC^MIN/+^ mice develop fewer intestinal adenomas and have larger spleens. (A) Representative ileal segments obtained from APC^MIN/+^ (CCR6WT- APC^MIN/+^) and CCR6KO- APC^MIN/+^ mice at 15 weeks of age magnified under a dissection microscope at 10X magnification. (B) Representative photomicrographs of hematoxylin and eosin stained slides of ileum collected from APC^MIN/+^ and CCR6KO- APC^MIN/+^ mice at 15 weeks of age. Tumors are marked by red dashed lines and normal epithelium is marked by green dashed lines (left panel). The regions in the squares are shown magnified (right panel). (C) Representative spleens from APC^MIN/+^ and CCR6KO-APC^MIN/+^ mice are shown in the left panel. Comparison of the mean spleen size for these two groups of mice (n = 8–13 per group) is shown in the right panel.

To address the role of CCL20-CCR6 interactions in intestinal tumorigenesis *in vivo*, we bred APC^MIN/+^ mice to CCR6-deficient (CCR6KO or CCR6^−/−^) mice to create APC^MIN/+^ mice lacking either one or both alleles of CCR6 (CCR6HET-APC^MIN/+^ or CCR6^+/−^-APC^MIN/+^ mice and CCR6KO-APC^MIN/+^ or CCR6^−/−^-APC^MIN/+^ mice respectively). APC^MIN/+^ mice have a single defective allele of *APC* and sporadically develop intestinal adenomas and early carcinomas [25], [26]. Phenotypically, CCR6KO-APC^MIN/+^ mice were seen to develop fewer intestinal adenomas (Figure 2A–B) and to have normal sized spleens compared to APC^MIN/+^ mice (Figure 2C). APC^MIN/+^ mice have large spleens associated with vigorous splenic hematopoiesis, possibly related to intestinal bleeding from the polyps [29].

**Figure 3:**
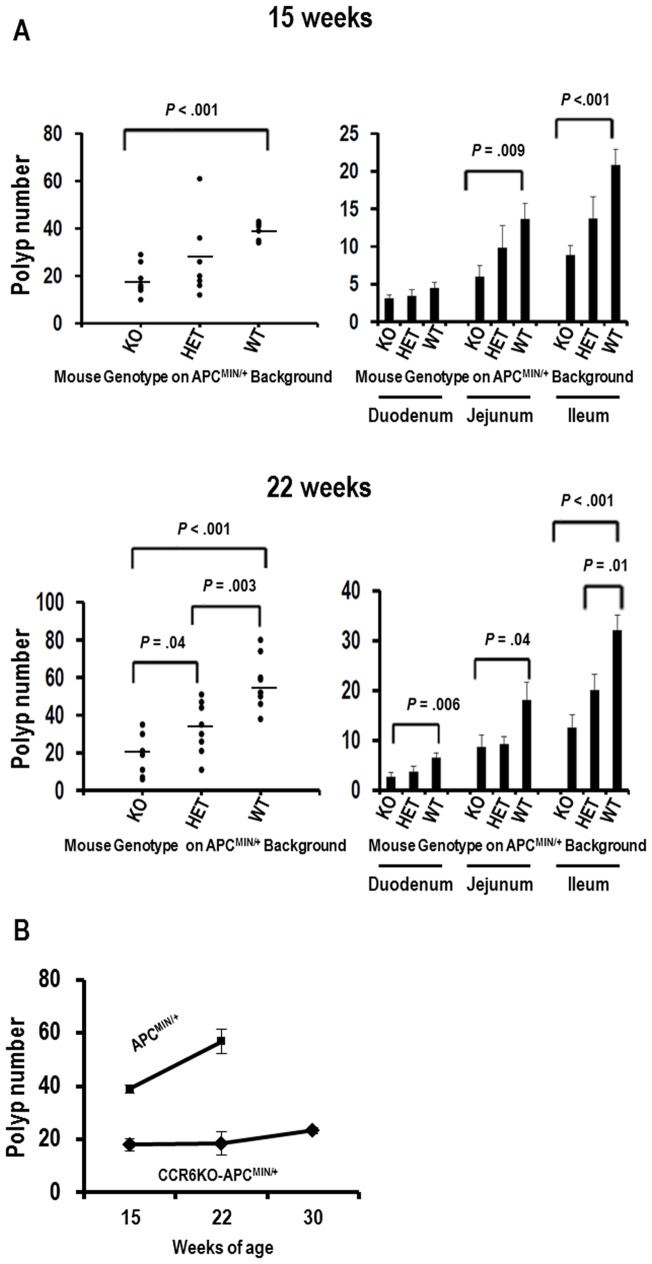
CCR6KO-APC^MIN/+^ mice develop fewer intestinal adenomas. (A) Polyp number is shown for the entire small intestine (left panels) and in the duodenum, jejunum and ileum individually (right panels) as compared between APC^MIN/+^, CR6HET- APC^MIN/+^, and CCR6KO- APC^MIN/+^ mice at 15 and 22 weeks of age (n = 6–9 mice per group at each time point). (B) Polyp number in the entire small intestine is shown for CCR6KO- APC^MIN/+^ and APC^MIN/+^ mice at 15, 22 and 30 weeks of age. APC^MIN/+^ mice could not be kept to 30 weeks of age as they uniformly reached endpoints for euthanasia.

The number of polyps and the size of each polyp in the small intestine were recorded for mice of 15 weeks and 22 weeks of age. At both time points, CCR6KO-APC^MIN/+^ mice had an approximately 2-fold decrease in the total number of intestinal tumors compared to that found in APC^MIN/+^ mice. The number of polyps in the CCR6HET-APC^MIN/+^ mice was intermediate (Figure 3A left panels). We then analyzed polyp number for each segment of the small intestine (Figure 3A right panels). APC^MIN/+^ mice develop the majority of the intestinal polyps in the ileum with the remaining polyps mostly arising in the jejunum. We found reduction of polyp number in all segments of the intestine for CCRKO-APC^MIN/+^ mice as compared to APC^MIN/+^ mice at both time points, although the reduction in the duodenum was not statistically significant at 15 weeks of age. We further quantified the burden of intestinal adenomas by calculating total polyp mass (Figure S1). Total polyp mass in the entire small intestine was approximately 3-fold lower in CCR6KO-APC^MIN/+^ mice as compared to APC^MIN/+^ mice at both the 15 and 22 week time points (Figure S1A left panels). Similarly, the polyp mass was decreased in all the segments of the small intestine in the CCR6KO-APC^MIN/+^ mice (Figure S1A right panels).

In our colony, APC^MIN/+^ mice almost uniformly meet endpoints for euthanasia by 25 weeks of age. In contrast, CCR6KO-APC^MIN/+^ mice were observed to be grossly healthy without any overt signs of distress at 30 weeks (Figure S2). Thus, we quantified adenoma burden in CCR6KO-APC^MIN/+^ mice at 30 weeks of age. At this time point, the polyp number was approximately half (Figure 3B) and the total polyp mass was approximately 2/3 (Figure S1B) of that seen in APC^MIN/+^ mice at 15 weeks of age.

### Decreased macrophage infiltration into adenomas and non-tumor epithelium in CCR6KO-APC^MIN/+^ mice as compared to APC^MIN/+^ mice without alterations in T cell, Treg, or B cell infiltration

**Figure 4:**
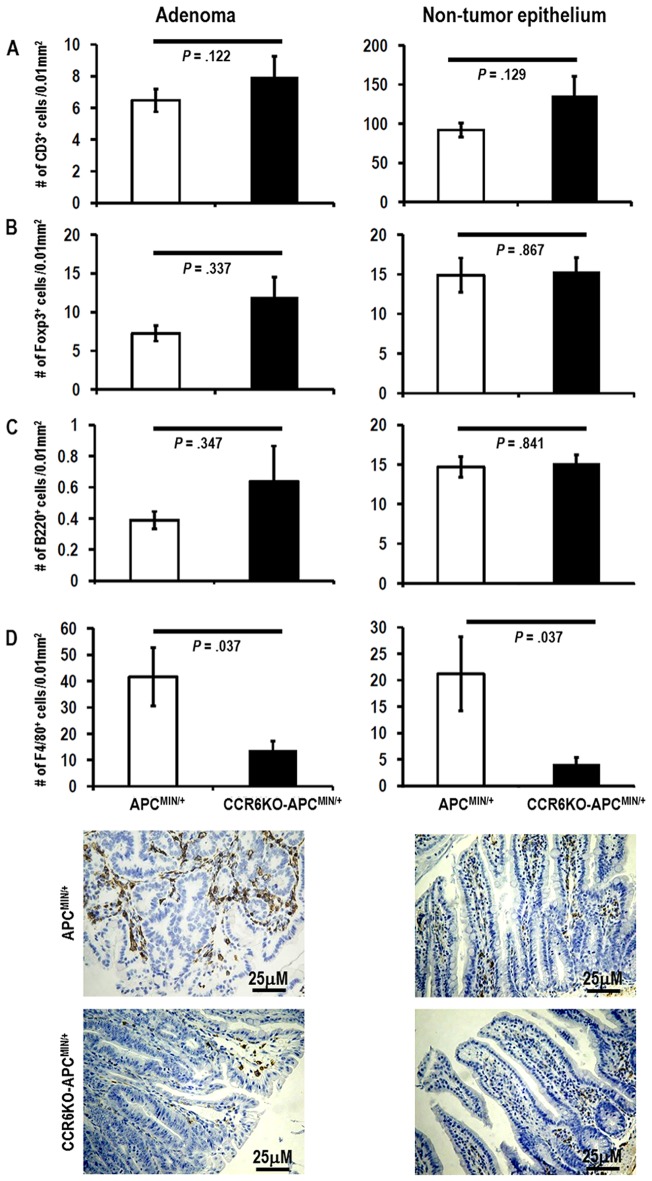
Macrophage infiltration into adenomas and non-tumor epithelium is decreased in CCR6KO-APC^MIN/+^ mice. Sections of paraffin-embedded immunohistochemically stained ileum from CCR6KO-APC^MIN/+^ and APC^MIN/+^ mice at 22 weeks of age were examined (n = 6–7 animals per group). Infiltrating (A) CD3^+^, (B) Foxp3^+^, (C) B220^+^, and (D top panels) F4/80^+^ cells were counted. Representative photomicrographs magnification showing macrophage infiltration in adenomas and normal epithelium in the ileum of APC^MIN/+^ and CCR6KO-APC^MIN/+^ mice are shown (D bottom panels).

Interactions between CCL20 and CCR6 are known to be particularly important for the migration of Th17 cells and Tregs into the intestine [30]. For these reasons, we suspected that alterations in the homing of immune cells might contribute to the decreased tumor formation in CCR6KO-APC^MIN/+^ mice. To explore this possibility, we evaluated the degree of infiltration of different immune cells (T cells, Tregs, B cells, and macrophages) *in situ* by immunohistochemically staining tissue sections of mouse ileum for CD3, FoxP3, B220, and F4/80 respectively and quantifying the number of stained cells in both the adenomas and non-tumor epithelium. We did not find significant differences in the infiltration of CD3^+^ cells, FoxP3^+^ cells or B220^+^ cells in either adenomas or non-tumor epithelium between CCR6KO-APC^MIN/+^ and APC^MIN/+^ mice as shown in Figure 4A–C (representative photomicrographs are shown in Figure S3). Counter-intuitively, in the absence of CCR6, there was a trend toward increased T cell infiltration into both adenomas and non-tumor epithelium. There was minimal infiltration of CD3^+^ cells, FoxP3^+^ cells, or B220^+^ cells in adenomas in either group when compared to adjacent non-tumor epithelium. Of note, we detected similar numbers of CD3^+^ cells and FoxP3^+^ cells in the adenomas in both groups suggesting that most of the T cells in the adenomas were Tregs. In contrast, only 16% and 10% of CD3^+^ T cells in the uninvolved epithelium were FoxP3^+^ cells in APC^MIN/+^ and CCR6KO-APC^MIN/+^ mice respectively. We did note a significant reduction in the number of F4/80^+^ cells in both tumors and uninvolved epithelium (Figure 4D) of the CCR6KO-APC^MIN/+^ mice. A significant number of macrophages were also found infiltrating human colorectal cancers (mean  = 36.1±3 cells/0.01 mm^2^, range  = 17.2–50.2) in the 11 tumors assessed as determined by immunohistochemical staining for CD163 (Figure S4) [31].

### CCL20 signaling through CCR6 promotes further production of CCL20 and induces proliferation of colorectal cells

**Figure 5:**
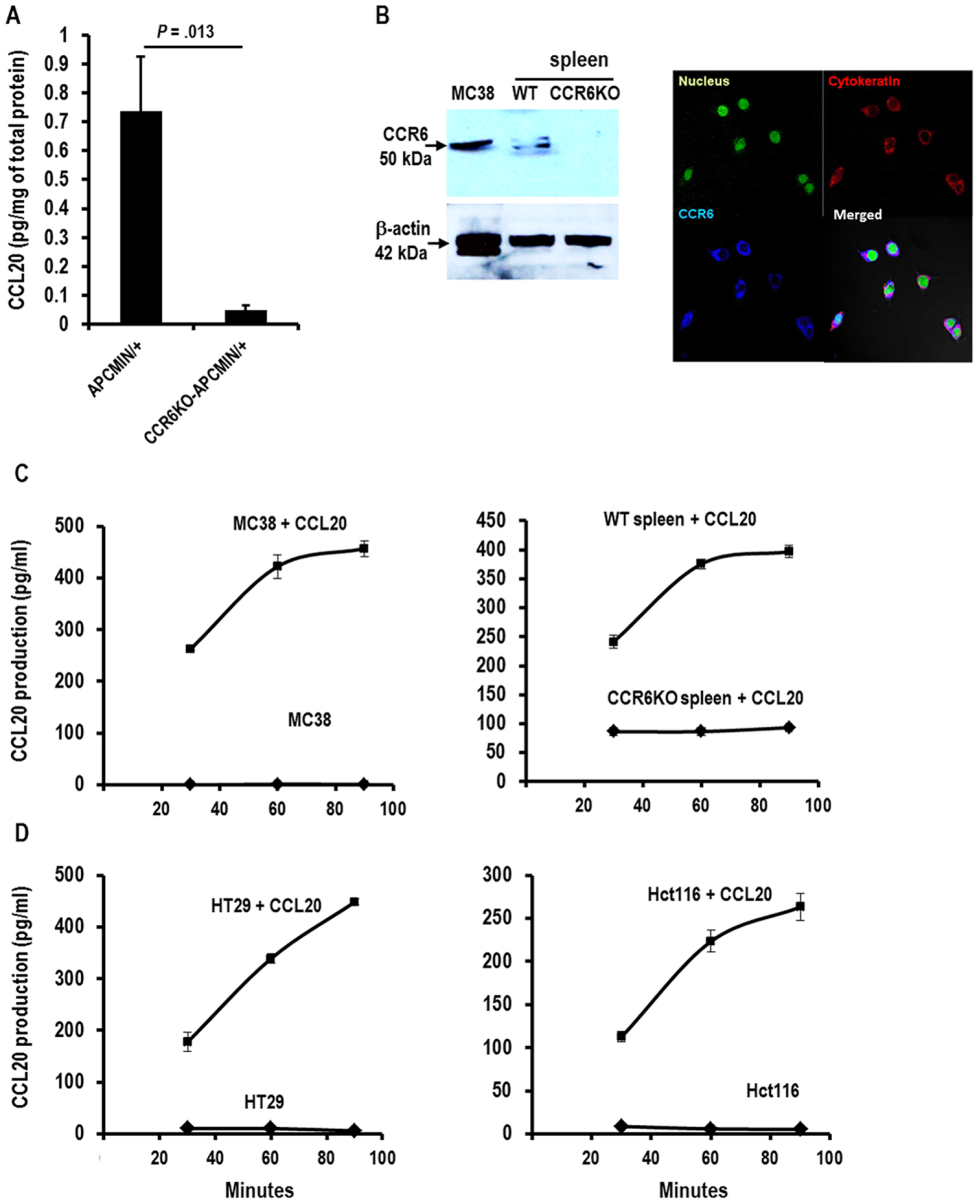
CCL20-CCR6 interactions induce the production of CCL20. (A) CCL20 concentrations in the ileum of APC^MIN/+^ and CCR6KO-APC^MIN/+^ mice at 15 weeks of age as measured by ELISA are shown. (Representative data from 1 of 2 experiments is shown.) (B) CCR6 expression is demonstrated in the murine colorectal cancer cell line MC38 both by Western blot analysis comparing cell lysates of MC38 cells, WT spleen, and CCR6KO spleen (left panel); and by immunofluorescence staining of cytospin specimens of MC38 cells (right panel). (C) CCL20-induced production of CCL20 was measured by exposing MC38 cells (left panel) as well as WT and CCR6KO splenocytes (right panel) to CCL20 at 50 ng/ml for 1 hour and analyzing culture supernatants 30, 60 and 90 minutes after withdrawal of CCL20. (Representative data from 1 of 3 experiments is shown.) (D) CCL20 was measured after exposing the human colon cancer cell lines HT29 (left panel) and Hct116 (right panel) to 50 ng/ml CCL20 for 1 hour and analyzing culture supernatants 30, 60 and 90 minutes after withdrawal of CCL20. (Representative data from 1 of 2 experiments is shown.).

We next assayed the levels of CCL20 in the ileum of APC^MIN/+^ and CCR6KO-APC^MIN/+^ mice at 15 weeks of age by subjecting homogenized tissue samples to ELISA. CCR6KO-APC^MIN/+^ mice were observed to have a significantly lower level of ileal CCL20 than that found in APC^MIN/+^ mice (Figure 5A). This finding implied that lack of CCR6 results in decreased production of CCL20. In order to determine whether signaling through CCR6 causes production of CCL20, we used ELISA to assay levels of CCL20 in the supernatant of MC38 murine colorectal cancer cells and splenocytes isolated either from wild-type or CCR6KO mice upon stimulation with CCL20. First, we checked for expression of CCR6 on the MC38 cell line. MC38 cell lysate was subjected to Western blot analysis with lysates of splenocytes from wild-type and CCR6KO mice used as positive and negative controls respectively. Our data demonstrate that CCR6 is expressed in this cell line (Figure 5B left panel). We next used immunofluorescence staining of cytospin samples of MC38 to confirm MC38 expression and determine its cellular location. Strong cell surface expression of CCR6 was observed (Figure 5B right panel). We then assayed for production of CCL20 by MC38 in response to CCL20 signaling. We pulsed MC38 cells with CCL20 for one hour, and then washed the cells and replaced the media. CCL20 concentration was assessed from 30 to 90 minutes after the stimulation. Stimulation of MC38 colorectal cancer cells with CCL20 resulted in a surge of CCL20 production at 30 minutes with a 2-fold increase by 60 minutes. CCL20 production appeared to plateau between 60 and 90 minutes (Figure 5C left panel). Next, we assayed CCL20 production in response to CCL20 in splenocytes from wild-type and CCR6KO mice to determine the role of CCR6 in CCL20-mediated CCL20 production. Wild-type splenocytes showed a 2-fold increase in CCL20 production compared to CCR6KO splenocytes 30 minutes after stimulation. Again, CCL20 production by wild-type splenocytes increased another 2-fold by 60 minutes, and production plateaued by 90 minutes (Figure 5C right panel). Similar kinetics of CCL20 production were demonstrated by the human colorectal cancer cell lines HT29 and Hct116 when exposed to CCL20 (Figure 5D). To exclude the possibility that the CCL20 expression we measured by ELISA in cells exposed to CCL20 was related to CCL20 dissociating from its receptor CCR6, we analyzed for *Ccl20* expression by quantitative reverse-transcription PCR. While the kinetics of C*cl20* expression after exposure to CCL20 varied between MC38, HT29, and Hct116, an approximately 2–6 fold increase in *Ccl20* was seen in these cell lines after stimulation with CCL20 (Figure S5A–C). We further demonstrated that the measured CCL20 expression was partially dependent on protein synthesis occurring after the CCL20 exposure by using cyclohexamide (a pan-protein synthesis inhibitor). MC38 cells were exposed to cyclohexamide for 4 hours prior to administration of CCL20, and CCL20 levels were then measured by ELISA. The presence of cyclohexamide significantly reduced the measured CCL20 at 60 and 90 minutes after withdrawal of CCL20 (Figure S5D). These experiments demonstrate that the specific interaction of CCL20 with CCR6 is responsible for auto-feedback loop regulation of CCL20 production.

**Figure 6:**
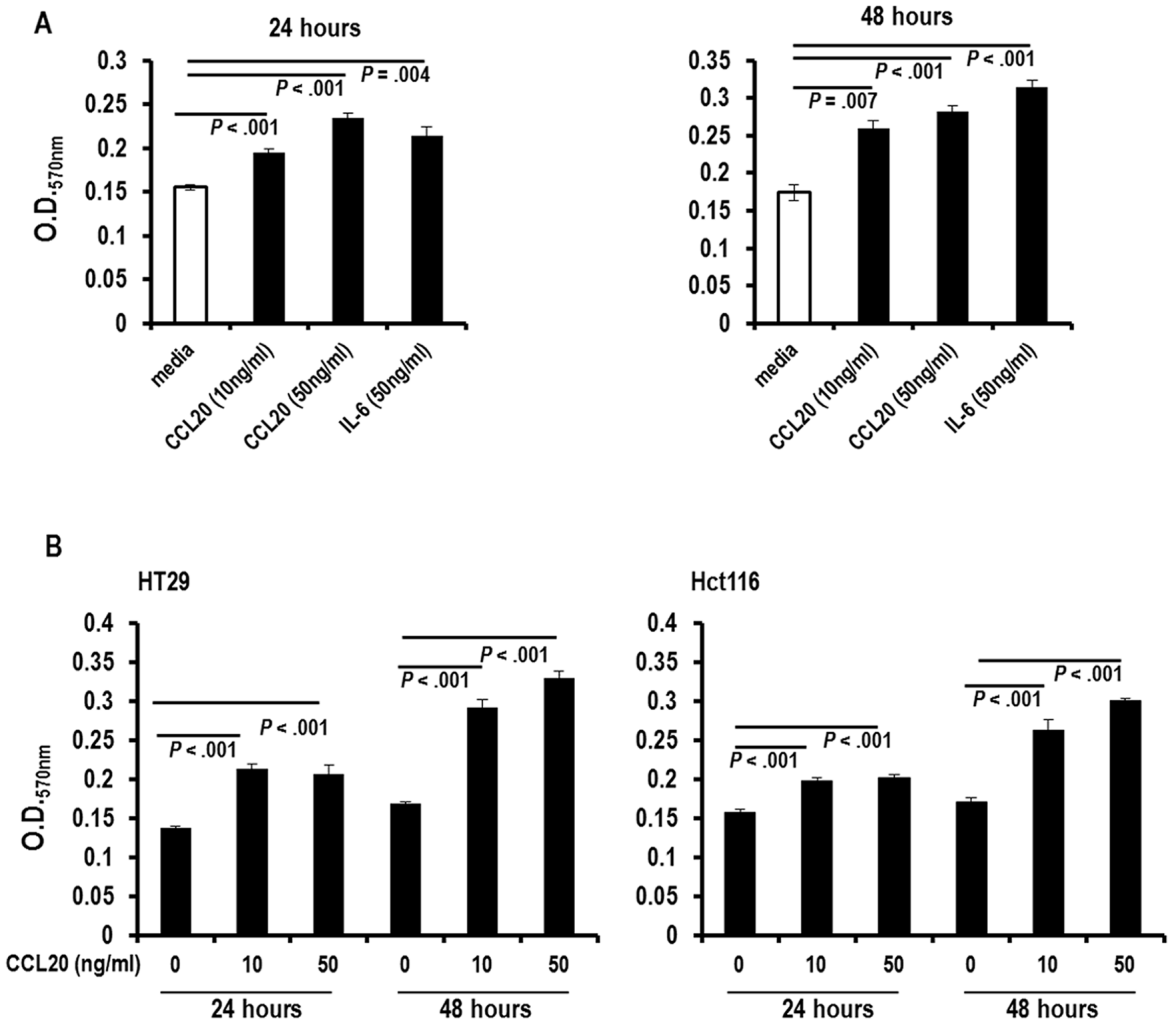
CCL20 induces proliferation in colon cancer cells. (A) Proliferation of MC38 cells after 24 and 48 hours of exposure to CCL20 at concentrations of 10 and 50 ng/ml, IL-6, or serum-free media as measured by the MTT assay is shown. (Representative data from 1 of 2 experiments is shown.) (B) Proliferation of HT29 (left) and Hct116 (right) cells after 24 and 48 hours of exposure to CCL20 at concentrations of 10 and 50 ng/ml. (Representative data from 1 of 2 experiments is shown.).

Having shown an auto-feedback loop in CCL20 production, we sought to confirm that CCL20 could induce proliferation of colorectal cancer cells. We subjected the MC38 cell line to the MTT assay after exposure to CCL20 or IL-6, which has previously been shown to increase proliferation of MC38 [32]. CCL20 induced a gradual increase in proliferation of MC38 cells in both a temporal- and concentration-dependent manner (Figure 6A). CCL20 similarly promoted proliferation in the human colorectal cancer cell lines HT29 and Hct116 (Figure 6B). The proliferative activity of CCL20 on the colorectal cancer cell lines was further confirmed using the ^3^H-thymidine incorporation assay (Figure S6).

## Discussion

The secretion of various soluble signaling molecules by resident stromal cells, infiltrating immune cells, and neoplastic tumor cells themselves is an important factor in tumor initiation and progression. Chemokines may play an important role in the crosstalk between immune cells and neoplastic tumor cells [1], [3] and as such may mediate the tumor promoting effects of inflammatory immune responses. In this study, we examined the role of the chemokine CCL20, which is the sole known ligand for the receptor CCR6, itself the sole known receptor for CCL20 [4], in intestinal tumorigenesis.

Recent evidence suggests a possible role for CCR6-CCL20 interactions in colorectal cancer. Expression of CCL20 and CCR6 has been observed in several malignancies including colorectal cancer [16]–[22]. A possible role of CCL20 in the liver mediating metastasis of CCR6-expressing colorectal cancer cells has been explored [16], [20], [21]. In fact, high serum levels of CCL20 have been shown to correlate with poor prognosis of the disease in human patients suggesting a possible causal role for CCL20-CCR6 interactions in promoting tumors [33]. Furthermore, direct effects of CCL20 in increasing proliferation [23], [24] and invasion [23] in colorectal cancer cell lines have been demonstrated *in vitro*. In addition to acting directly on neoplastic tumor cells, it is also conceivable that CCL20-CCR6 interactions promote cancer through their role in inflammation. CCL20 and CCR6 have been shown to be important mediators of various inflammatory [5]–[9]. Inflammation is now recognized as playing an important role in sporadic intestinal tumorigenesis [10]–[15], and this association has been repeatedly demonstrated in the APC^MIN/+^ mouse model.[10], [12], [13], [15].

The primary aim of this study was to evaluate the role of the disruption of CCL20-CCR6 interactions in a model of sporadic intestinal tumorigenesis. While blockade of CCL20-CCR6 interactions has been shown to decrease colorectal tumor growth in a xenograft model [28] and administration of exogenous CCL20 has been shown to promote growth of syngeneic transplanted colorectal tumors [34], to our knowledge the effect of blockade of CCL20-CCR6 interactions on intestinal carcinogenesis *in vivo* in the setting of an intact host immune system has not been reported.

Our data clearly show that CCR6 is an important factor in the sporadic development of intestinal adenomas as its absence results in a marked decrease in adenomas by 2 measures at 2 time points. The decrease was noted in each segment of the small intestine in the APC^MIN/+^ mouse model. We further noted that APC^MIN/+^ mice without functional CCR6 appeared to live longer and to be healthy at a later time point due to the decreased adenoma development. CCR6KO-APC^MIN/+^ mice were observed to have smaller spleens than APC^MIN/+^ mice. Adenoma-bearing APC^MIN/+^ mice have characteristically larger spleens as compared to non-adenoma bearing wild-type mice. This has been attributed to extramedullary hematopoiesis, as these animals are typically anemic due to the intestinal adenomas [29]. It is also possible that the enlarged spleens are partially related to an altered immune system in APC^MIN/+^ mice [10] but in either case, spleen size in APC^MIN/+^ mice typically correlates with the burden of intestinal adenomas.

We also made interesting initial observations on the potential mechanism of CCR6 in adenoma development. We noted decreased infiltration of F4/80^+^ macrophages in adenomas and non-adenoma intestinal epithelium of CCR6KO-APC^MIN/+^ mice suggesting a role for CCL20-CCR6 interactions in macrophage infiltration. Indeed CCL20 (also known as macrophage inflammatory protein-3α) has been shown to play an important role in the maturation of intestinal macrophages [35] and in the migration of macrophage-lineage cells such as Langerhans cells [36] and dendritic cells [37]. A tumor promoting role of tumor associated macrophages (TAMs) has been demonstrated for many cancer types in many models [38]. Interestingly, a role for macrophages in promoting colorectal cancer has been noted by a recent paper where the effect appeared to be at least partially due to the loss of macrophage-generated CCL20 [34]. The role for macrophages in intestinal tumorigenesis was also supported by another recent paper demonstrating that knockout of monocyte chemoattractant protein 1 (MCP-1) in APC^MIN/+^ mice is associated with decreased macrophages in intestinal adenomas and non-adenoma intestinal epithelium as well as decreased adenoma formation [39]. Another recent study demonstrated that myeloid cells, particularly Gr1^+^ but also F4/80^+^ and CD11b^+^ cells, respond to intestinal microbiota by secreting IL-23, which is particularly important in the development of adenomas in the colon of mice with an APC mutation [11].

In contrast, we did not notice a decrease in T cell number in the adenomas or non-adenoma intestinal epithelium of CCR6KO-APC^MIN/+^ mice as compared to APC^MIN/+^ mice, and in fact there was a trend toward an increase in T cell infiltration. Similarly, we did not notice differences in the infiltration of B cells or Tregs (regulatory T cells). These results are in line with reports from other groups showing an apparent increase in T cell infiltration and no change in B cell infiltration in the intestinal epithelium of CCR6KO mice [40], [41]. Of note, another group found that Treg migration into colorectal cancer was dependent on CCL20-CCR6 signaling for tumors induced by the carcinogen N-methyl-N-nitrosourea (MNU) or the bacterium *H. pylori* as well as in a syngeneic transplantable tumor model [34]. Perhaps, the influence of CCL20-CCR6 interactions on Treg migration into colorectal cancer is dependent on the model. It is possible, for instance, that Treg trafficking to colorectal cancer is controlled differently than to adenomas. It is also possible that the difference could be explained by the fact that our study employed a sporadic carcinogenesis model as opposed to models where tumors are induced under inflammatory conditions or transplanted heterotopically into mice. Interestingly, in our study it appears that the vast majority of the CD3^+^ T cells in adenomas of APC^MIN/+^ mice are Tregs as determined by expression of FoxP3.

Our data also showed that there is less CCL20 in the small intestine of CCR6KO-APC^MIN/+^ mice as compared to APC^MIN/+^ mice. This observation prompted us to investigate if CCL20 signaling through CCR6 promotes further secretion of CCL20. We demonstrated that this indeed was the case using a murine colorectal cancer cell line and 2 human colorectal cell lines as well as murine splenocytes. We further confirmed that CCL20 induces proliferation in the murine and human colorectal cancer cell lines as has been shown by other groups for human colorectal cancer cell lines [23], [24].

Further studies, however, will be required to determine the precise mechanism by which CCL20-CCR6 interactions promote intestinal tumorigenesis. The relative role of direct effects on neoplastic epithelial cells as compared to effects on stromal cells such as macrophages remains to be elucidated.

In summary, loss of CCR6 disrupts the formation of intestinal adenomas in a murine model of intestinal carcinogenesis. Loss of CCR6 is also associated with decreased CCL20 levels in the intestine of this mouse model as CCL20-CCR6 interactions promote further secretion of CCL20. Disruption of CCL20-CCR6 interactions results in decreased macrophage migration as well as decreased proliferation of neoplastic epithelial cells. The relative contributions of these 2 effects to decreased tumorigenesis as well as the mechanism by which these effects are mediated remain to be elucidated.

Our results suggest an important role of CCL20-CCR6 in intestinal neoplasia. The role of CCL20-CCR6 interactions in tumor development, however, is likely not restricted to this model. Associations between CCL20-CCR6 interactions and many cancer types have been suggested. As such, blockade of this interaction warrants further investigation as a potential target for the treatment and prevention of malignancy.

## Supporting Information

Figure S1 **CCR6KO-APC^MIN/+^ mice develop reduced polyp mass.** (A) Polyp mass is shown for the entire small intestine (left panels) and the duodenum, jejunum and ileum individually (right panels) as compared between APC^MIN/+^, CR6HET- APC^MIN/+^, and CCR6KO- APC^MIN/+^ mice at 15 and 22 weeks of age (n = 6–9 mice per group at each time point). (B) Polyp mass in the entire small intestine is shown for CCR6KO- APC^MIN/+^ and APC^MIN/+^ mice at 15, 22 and 30 weeks of age. APC^MIN/+^ mice could not be kept to 30 weeks of age as they uniformly reached endpoints for euthanasia.
(TIF)

Figure S2 **CCR6KO-APC^MIN/+^ mice grossly appear healthy up to 30 weeks of age.** Representative pictures of APC^MIN/+^ and CCR6KO-APC^MIN/+^ of 22 weeks and 30 weeks of age.
(TIF)

Figure S3 **T cell, Treg, or B cell infiltration is unchanged between CCR6KO-APC^MIN/+^ mice and APC^MIN/+^ mice.** Representative photomicrographs of sections of paraffin-embedded ileum from APC^MIN/+^ (top panels) and CCR6KO-APC^MIN/+^ (bottom panels) mice at 22 of weeks age immunohistochemically stained for (A) CD3, (B) Foxp3, and (C) B220 cells are shown in adenoma (left panel) and normal epithelium (right panel).
(TIF)

Figure S4 **Infiltration of macrophages in human colorectal cancer.** A representative photomicrograph of a paraffin-embedded section of colorectal cancer immunohistochemically stained with an antibody specific for CD163 is shown.
(TIF)

Figure S5 **Exposure to CCL20 induces expression of** ***Ccl20*** **and CCL20 protein synthesis.** *Ccl20* expression as measured by quantitative RT-PCR after exposure to 50 ng/ml CCL20 for 1 hour and isolation of RNA at 30, 60 and 90 minutes following withdrawal of CCL20 is shown for MC38 (A), HT29 (B) and Hct116 (C) cell lines. Relative expression was normalized to *gapdh* and calculated using the 2^−ΔΔCt^ method. (Representative data from 1 of 2 experiments is shown.) (D) CCL20-induced production of CCL20 in MC38 cells either with or without cyclohexamide treatment (5 µg/ml for 4 hours prior to CCL20 exposure) was measured after exposing cells to CCL20 at 50 ng/ml for 1 hour and analyzing culture supernatants 30, 60 and 90 minutes after withdrawal of CCL20. * p<.05.
(TIF)

Figure S6 **CCL20 induces proliferation in colon cancer cells.** Proliferation of MC38, HT29 and Hct116 cells measured by ^3^H-thymidine incorporation assay after 48 hours of exposure to CCL20 at 50 ng/ml. Data are shown as percentage increase in proliferation compared to cells cultured in the absence of CCL20. * p<.05.
(TIF)

Methods S1 **Supplementary Materials and Methods.**
(DOC)
