# Supplementary figures and images for: CCR6, the Sole Receptor for the Chemokine CCL20, Promotes Spontaneous Intestinal Tumorigenesis
Source: PLoS One. 2014 May 27;9(5):e97566. doi: 10.1371/journal.pone.0097566 (PMC4035256; doi:10.1371/journal.pone.0097566)

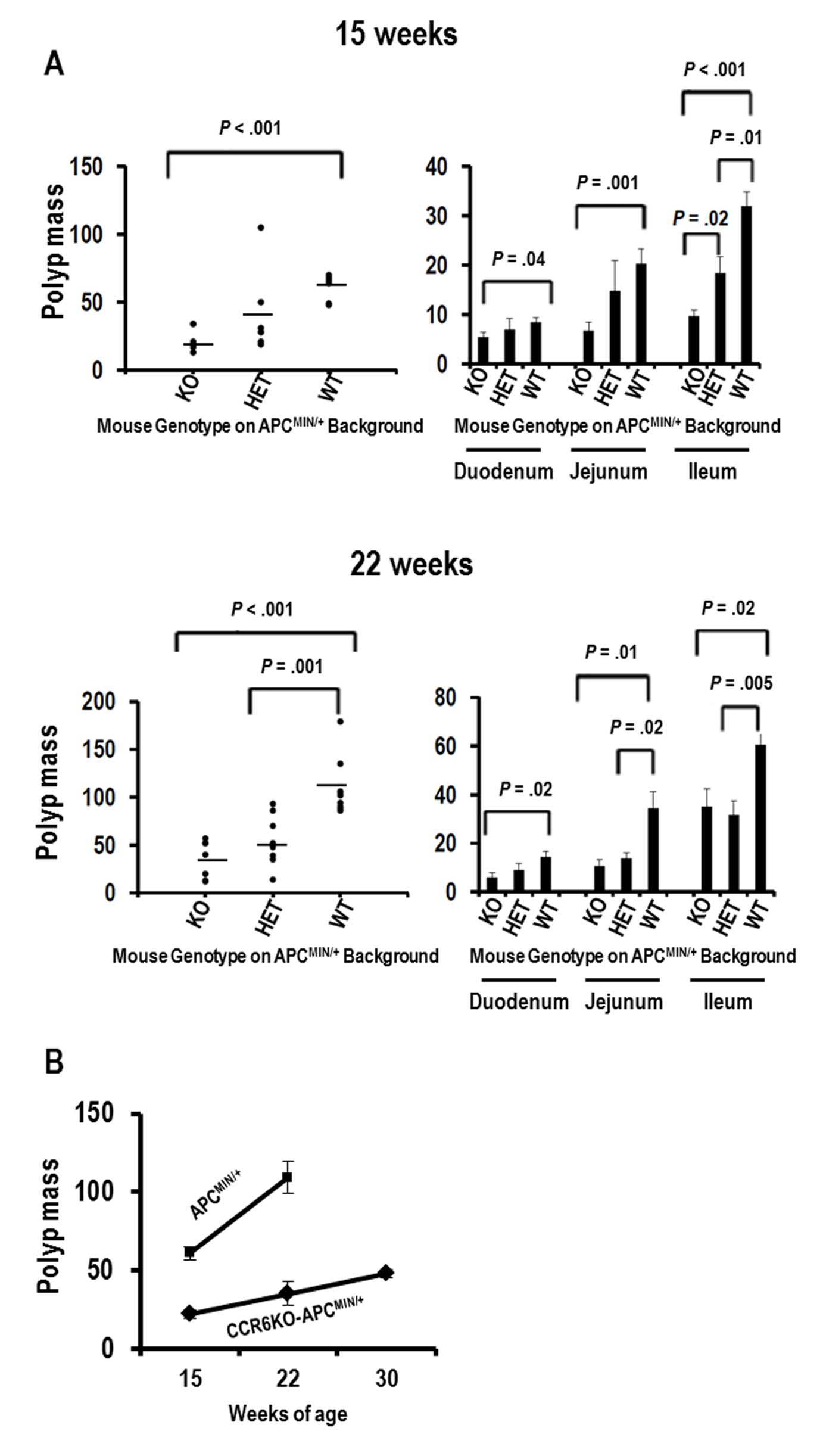

Supplement: Figure S1 — CCR6KO-APCMIN/+ mice develop reduced polyp mass. (A) Polyp mass is shown for the entire small intestine (left panels) and the duodenum, jejunum and ileum individually (right panels) as compared between APCMIN/+, CR6HET- APCMIN/+, and CCR6KO- APCMIN/+ mice at 15 and 22 weeks of age (n = 6–9 mice per group at each time point). (B) Polyp mass in the entire small intestine is shown for CCR6KO- APCMIN/+ and APCMIN/+ mice at 15, 22 and 30 weeks of age. APCMIN/+ mice could not be kept to 30 weeks of age as they uniformly reached endpoints for euthanasia. (TIF) [file pone.0097566.s001.tif]

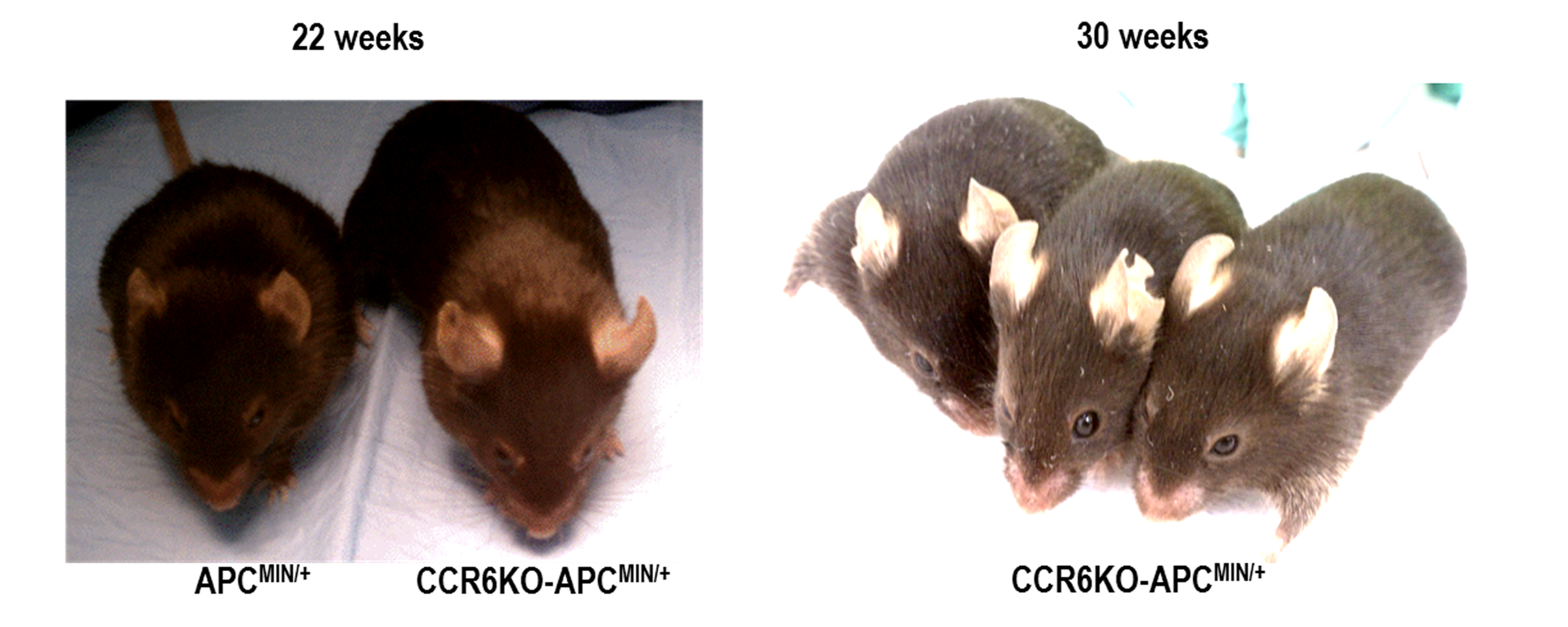

Supplement: Figure S2 — CCR6KO-APCMIN/+ mice grossly appear healthy up to 30 weeks of age. Representative pictures of APCMIN/+ and CCR6KO-APCMIN/+ of 22 weeks and 30 weeks of age. (TIF) [file pone.0097566.s002.tif]

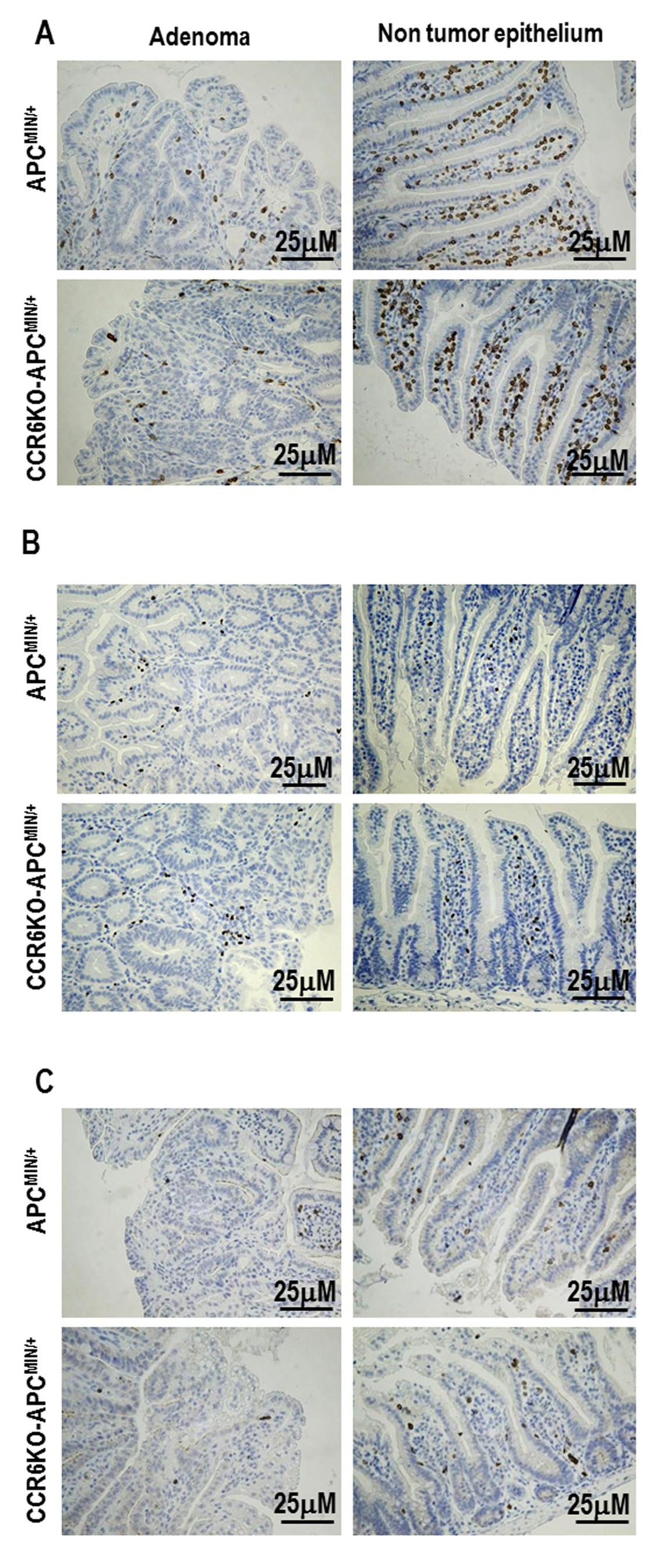

Supplement: Figure S3 — T cell, Treg, or B cell infiltration is unchanged between CCR6KO-APCMIN/+ mice and APCMIN/+ mice. Representative photomicrographs of sections of paraffin-embedded ileum from APCMIN/+ (top panels) and CCR6KO-APCMIN/+ (bottom panels) mice at 22 of weeks age immunohistochemically stained for (A) CD3, (B) Foxp3, and (C) B220 cells are shown in adenoma (left panel) and normal epithelium (right panel). (TIF) [file pone.0097566.s003.tif]

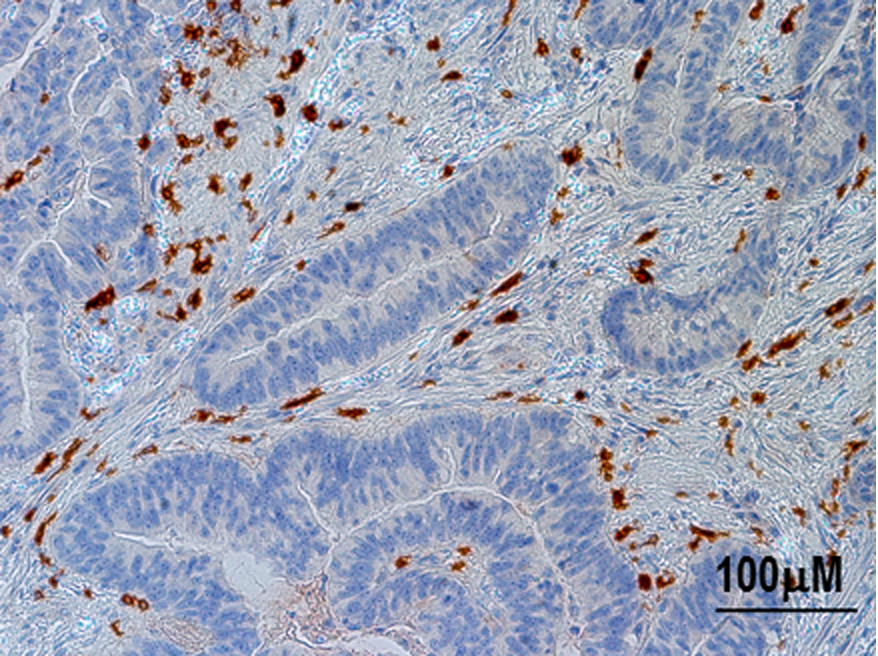

Supplement: Figure S4 — Infiltration of macrophages in human colorectal cancer. A representative photomicrograph of a paraffin-embedded section of colorectal cancer immunohistochemically stained with an antibody specific for CD163 is shown. (TIF) [file pone.0097566.s004.tif]

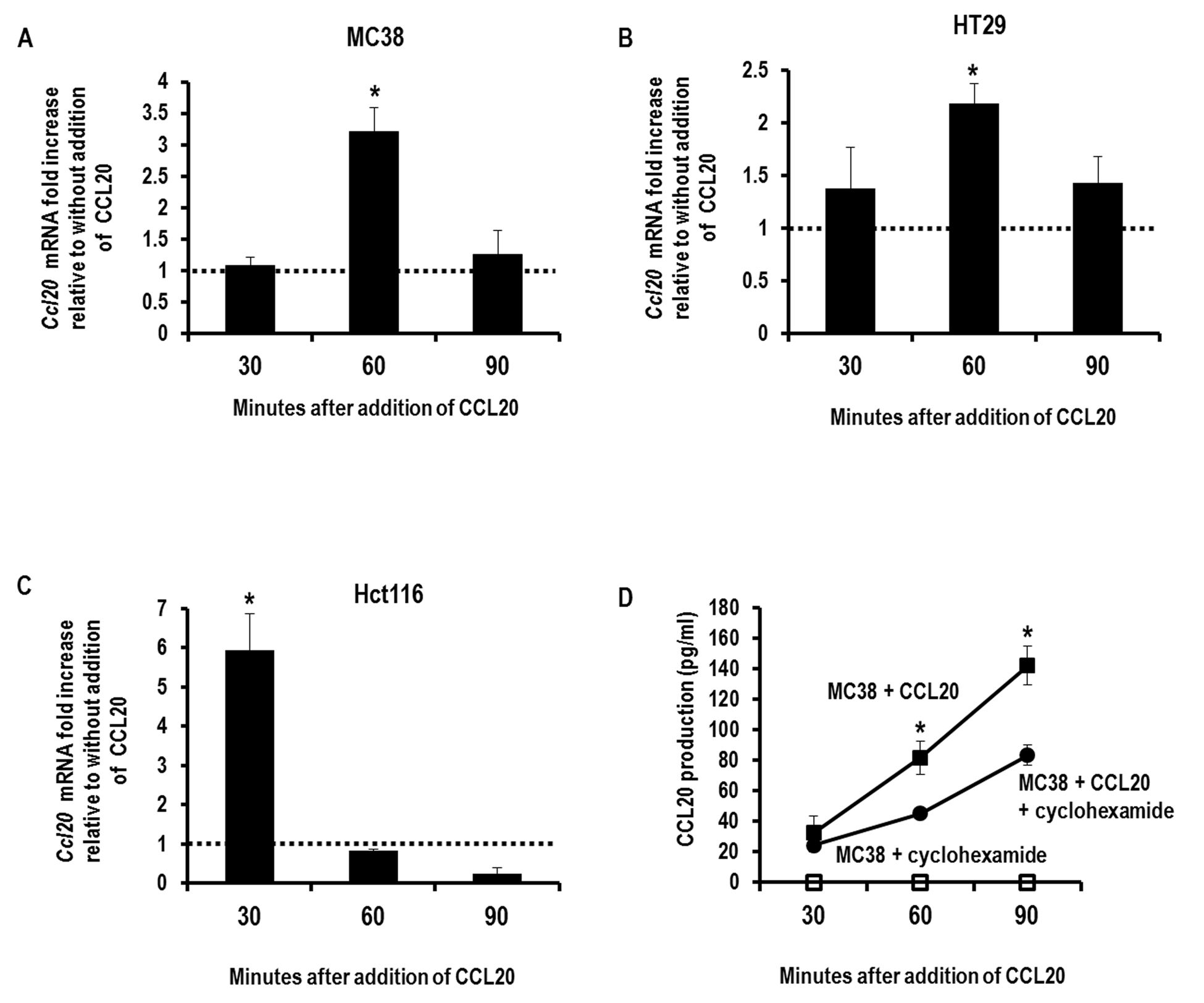

Supplement: Figure S5 — Exposure to CCL20 induces expression of Ccl20 and CCL20 protein synthesis. Ccl20 expression as measured by quantitative RT-PCR after exposure to 50 ng/ml CCL20 for 1 hour and isolation of RNA at 30, 60 and 90 minutes following withdrawal of CCL20 is shown for MC38 (A), HT29 (B) and Hct116 (C) cell lines. Relative expression was normalized to gapdh and calculated using the 2−ΔΔCt method. (Representative data from 1 of 2 experiments is shown.) (D) CCL20-induced production of CCL20 in MC38 cells either with or without cyclohexamide treatment (5 µg/ml for 4 hours prior to CCL20 exposure) was measured after exposing cells to CCL20 at 50 ng/ml for 1 hour and analyzing culture supernatants 30, 60 and 90 minutes after withdrawal of CCL20. * p<.05. (TIF) [file pone.0097566.s005.tif]

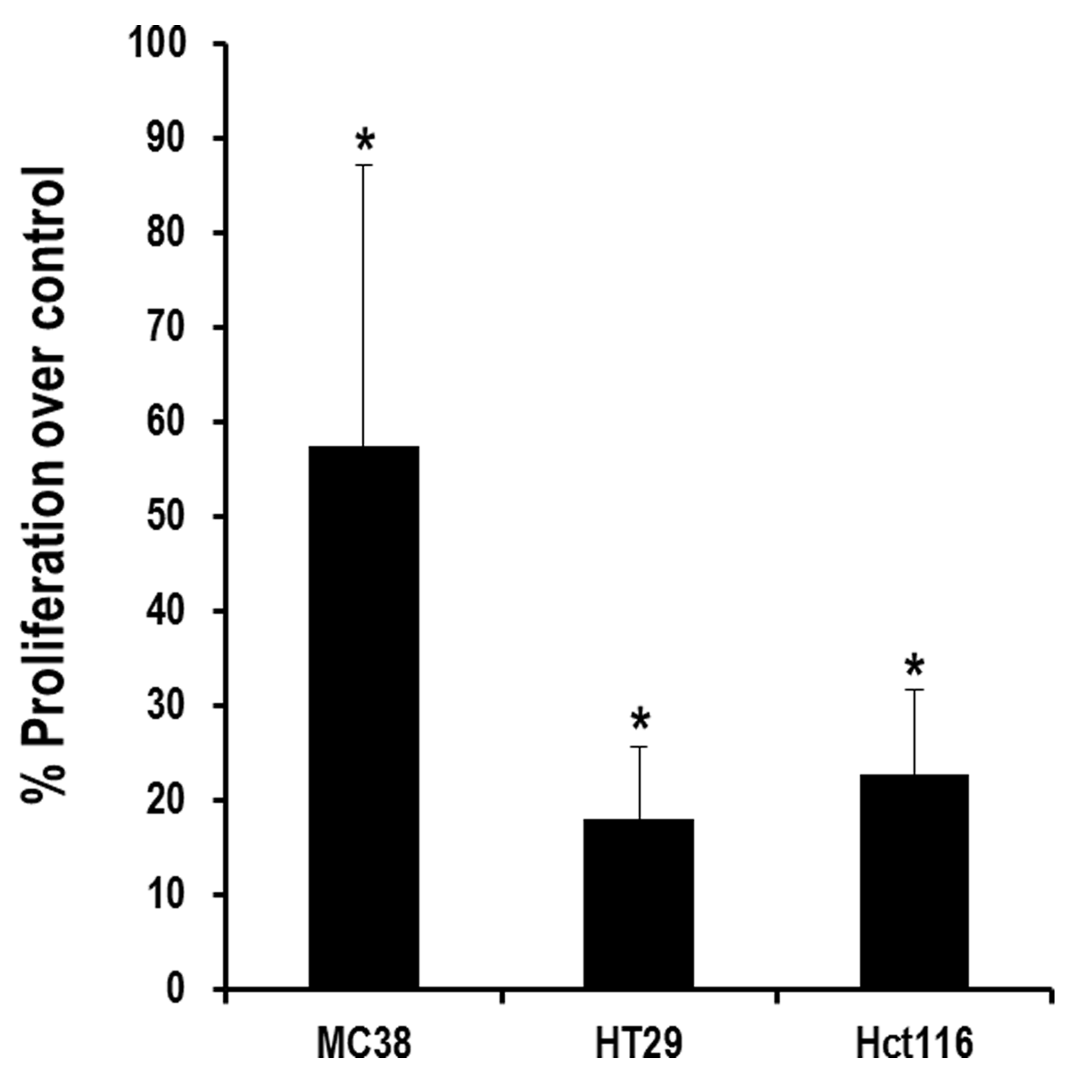

Supplement: Figure S6 — CCL20 induces proliferation in colon cancer cells. Proliferation of MC38, HT29 and Hct116 cells measured by 3H-thymidine incorporation assay after 48 hours of exposure to CCL20 at 50 ng/ml. Data are shown as percentage increase in proliferation compared to cells cultured in the absence of CCL20. * p<.05. (TIF) [file pone.0097566.s006.tif]
