# Supplementary material for: CCR6, the Sole Receptor for the Chemokine CCL20, Promotes Spontaneous Intestinal Tumorigenesis
Source: PLoS One. 2014 May 27;9(5):e97566. doi: 10.1371/journal.pone.0097566 (PMC4035256; doi:10.1371/journal.pone.0097566)
Supplement: Methods S1 — Supplementary Materials and Methods. (DOC) [file pone.0097566.s007.doc]

**Supplementary Materials and Methods**

**Immunohistochemistry:** Paraffin-embedded sections were heated at 58°C for 1 hour and deparaffinized by washing twice with Xylene for 5 minutes followed by washing in 100% and 80% ethanol sequentially, twice each for 5 minutes. The slides were then rinsed with water. Next, the sections were subject to antigen retrieval by placing the slides in either a pH 6 or pH 8 citrate buffer or a pH 8 EDTA buffer with or without proteinase K (all from Life Technologies), depending on the primary antibody and heating them in a pressure cooker (Biocare Medical, Concord, CA) at 125°C for 30 seconds. After cooling, the slides were washed with Tris buffered saline (TBS) with Tween 20 (Dako), dried in a humidifier chamber, blocked with peroxide block (Dako) for 5 minutes, and then blocked with protein block (Dako) for 20 minutes. Next, the sections were immersed with primary antibody in the appropriate dilution of antibody diluent buffer (Dako) for 1 hour, washed with TBS, and then bathed in TBS for 5 minutes. Next the sections were treated with secondary antibody (EnVision anti-rabbit or EnVision anti-mouse, Dako) for 30 minutes. After further washing, the sections were developed with one drop of diamino benzidine (DAB) chromogen-substrate. Slides were then washed with water and counterstained with hematoxylin for 1 minute. The conditions for each primary antibodies were as follows: anti-mouse B220 - pH 6 citrate retrieval, 1:200 dilution; anti-mouse CD3 - pH 8 EDTA retrieval, 1:300 dilution; anti-mouse F4/80 - pH 8 EDTA with proteinase K retrieval, 1:10,000 dilution; anti-mouse FoxP3 - pH6 citrate retrieval, 1:12 dilution; anti-human CD163 - pH6 citrate retrieval, 1:250 dilution; anti-human CCR6 - pH6 citrate retrieval, 1:100 dilution; anti-human CCL20 - pH 8 EDTA retrieval, 1:50 dilution.

**Semi-quantitative RT-PCR and real-time RT-PCR:** The following primers were used for semi-quantitative RT-PCR for *CCR6* . *β-actin* forward: 5′-CCCTGGACTTCGAGCAAGAG-3’, reverse: 5′-TCTCCTTCTGCATCCTGTCG-3′;  *Ccl20* forward: 5’-ATGTGCTGTACCAAGAGTTT-3’, reverse: 5′-CAAGTCTGTTTTGGATTTGC-3′;  *Ccr6* forward: 5′-CCATTCTGGGCAGTGAGTCA-3′, reverse: 5′-AGCAGCATCCCGCAGTTAA-3′. The PCR conditions were as follows: melting at 94⁰C for 30 seconds, annealing at 58⁰C for 1 minute, and extension at 72⁰C for 45 seconds for 34 cycles. Upon completion of PCR, the amplicons were run on 2% agarose gel and band intensities for both *β-actin* and *Ccr6* were compared using ImageJ software (National Institute of Health, Bethesda, MD).

cDNA from MC38, HT29 and Hct116 cells was used to quantitate  *Ccl20* expression in relation to the housekeeping gene *glyceraldehyde 3-phosphate dehydrogenase* (*gapdh*) with real-time RT-PCR. The following PCR primers were used [1]. Human  *Ccl20* forward: 5′-CTGGCTGCTTTGATGTCAGT-3′, reverse: 5′-CGTGTGAAGCCCACAATAAA-3′; mouse  *Ccl*20 forward: 5′-GTGGGTTTCACAAGACAGATG-3′, reverse: 5′-TTTTCACCCAGTTCTGCTTTG-3′; human *gapdh* forward: 5′-CAATGACCCCTTCATTGACC-3′, reverse: 5′-GACAAGCTTCCCGTTCTCAG-3′ and mouse *gapdh* forward: 5′-TGTGTCCGTCGTGGATCTGA-3′, reverse: 5′-CCTGCTTCACCACCTTCTTGAT-3′. 2 μl of cDNA and 0.5 μM of each primer were mixed with 25 μl of 2x Power SYBR Green PCR Master Mix (Life Technologies) to a final reaction volume of 50 μl. All reactions were run in triplicate in 96-well optical reaction plates (Life Technologies) using the ABI PRISM 7900HT Sequence Detection System (Life Technologies) with the following conditions: 95⁰C for 10 min for initial melting followed by 40 cycles of 95⁰C melting for 10 sec and 60⁰C annealing and extension for 1 minute. Relative expression was normalized to *GAPDH* and calculated using the 2^- ΔΔCt^ method. Results were expressed in fold change.

**^3^H-thymidine proliferation assay:** Three different cell lines (MC38, HT29 and Hct116) were cultured in triplicate in serum free RPMI 1640 medium for 48 hours in the presence or absence of CCL20 at a concentration of 50ng/ml. Proliferation was assessed by 3H-thymidine incorporation over 6 hours. The radioactivity was measured in a liquid scintillation counter

**Reference**

1. Kao CY, Huang F, Chen Y, Thai P, Wachi S, et al. (2005) Up-regulation of CC chemokine ligand 20 expression in human airway epithelium by IL-17 through a JAK-independent but MEK/NF-kappaB-dependent signaling pathway. J Immunol 175: 6676-6685.
